# Transcriptional activation of the human papillomavirus type 5 and 16 long control region in cells from cutaneous and mucosal origin

**DOI:** 10.1186/1743-422X-4-27

**Published:** 2007-03-12

**Authors:** Nitesh Mistry, Monika Simonsson, Magnus Evander

**Affiliations:** 1 Department of Virology, Umeå University, S-901 85 Umeå, Sweden

## Abstract

Human papillomavirus type-16 (HPV-16) infects mucosal epithelium and is the most common type found in cervical cancer. HPV-5 infects cornified epithelium and is the most common type found on normal skin and belongs to the types frequently associated with skin cancers of *Epidermodysplasia verruciformis *patients. One factor by which this anatomical tropism could be determined is the regulation of HPV gene expression in the host cell. The HPV long control region (LCR) contains cis-responsive elements that regulate HPV transcription and the epithelial tropism of HPV is determined by epithelial specific constitutive enhancers in the LCR. Since HPV-16 and other types infecting the mucosa differ in host cell from HPV types infecting skin, it has been hypothesized that it is the combination of ubiquitous transcription factors working in concert in the host cell that determines the cell-type-specific expression. To study if HPV tropism could be determined by differences in transcriptional regulation we have cloned the transcriptional regulating region, LCR, from HPV-16 and HPV-5 and studied the activation of a reporter gene in cell lines with different origin. To analyse promoter activity we transfected the plasmids into four different cell lines; HaCaT, C33A, NIKS and W12E and the efficiency of HPV-5 and HPV-16 LCR in the different cell lines was compared. In HaCaT cells, with a skin origin, the HPV-5 LCR was two-fold more efficient in transcriptional activation compared to the HPV-16 LCR. In cervical W12E cells the HPV-16 LCR was almost 2-fold more effective in activating transcription compared to the HPV-5 LCR. The ability to initiate transcription in the other cell lines was independent on cell origin and HPV-type.

## Findings

Papillomaviruses belong to the *Papillomaviridae *family and consist of a large family of non-enveloped double stranded DNA viruses that infect the cutaneous or mucosal epithelia of a dozen vertebrate species with a strict species tropism. Over 100 human papillomavirus (HPV) types have been completely described and identified in human tissues and they together with animal papillomaviruses are divided into 18 genera based on their nucleotide sequence identity of the major capsid protein L1 open reading frame (ORF) [1]. HPV-16 infects mucosal cells and belongs to the alpha-papillomavirus genus, species 9 [1] and is the most common type found in cervical cancer but does also cause condyloma and other infections of the genital, oral and respiratory tracts [2]. HPV-5 infects skin and is the most common type found on normal skin all around the world [3]. It belongs to species 1 of the beta-papillomavirus genus [1] and is associated with skin cancers in patients diagnosed with *Epidermodysplasia verruciformis*. All HPVs cause lesions of epithelial origin, but nothing is known regarding why certain HPVs are only detected in skin while some are only found in mucosal cells. It has been suggested that the different biology of transcriptional regulation of alpha and beta HPVs could be important for this tropism [4].

Once HPV has entered the cell, the viral DNA is transported to the nucleus for transcription and replication. The long control region (LCR) of HPV contains enhancer elements responsive to cellular factors and virally encoded transcriptional regulatory factors and is positioned between the L1 and E6 gene. The transcription is tightly regulated by the differentiation state of the infected epithelial cell [5-9] and the location of transcription initiation is found in the LCR. All papillomavirus LCRs studied so far contain epithelial specific constitutive enhancers [10] that contribute to the epithelial tissue tropism of HPVs. Most HPV-types have a promoter in front of the E6 gene in common [11,12]. For HPV-16, P_97 _is the major promoter which directs the expression of E6 and E7 as well as several other early gene products. It is analogous to the P_97 _of HPV-31 and P_105 _of HPV-18. For HPV-5, two promoters are suggested to exist within the LCR, an early E6 promoter and a late promoter [13]. Their positions are most probably similar to the closely related HPV-8 promoters P_175 _and P_7535 _[12,14]. In alpha HPVs transcription starts with the interaction of transcription factors with the TATA-box and Sp1 binding site on E6 promotor [15]. Depending on the HPV type these enhancers differ in quantity, arrangement and type. A synergism between factors like AP-1, NF-1, TEF-1 and Sp1 and the composition of their subunits seem to play an important role for HPV transcription in the epithelial cell layers where HPV is actively transcribed [16-19].

Since mucosal and cutaneous epithelium has been shown to express different proteins [20] it is conceivable that cutaneous HPV-5 and mucosal HPV-16 are exposed to a different transcriptional environment. The HPV LCR may also have different capacity to cooperate with cellular factors from the host cell depending on HPV type and cell line origin. To study if tropism could in part be determined by differences in transcriptional regulation we have cloned the transcriptional regulating region, LCR, from HPV-5 and HPV-16 and studied the activation of a reporter gene in cell lines with different origin.

**Figure 1:**
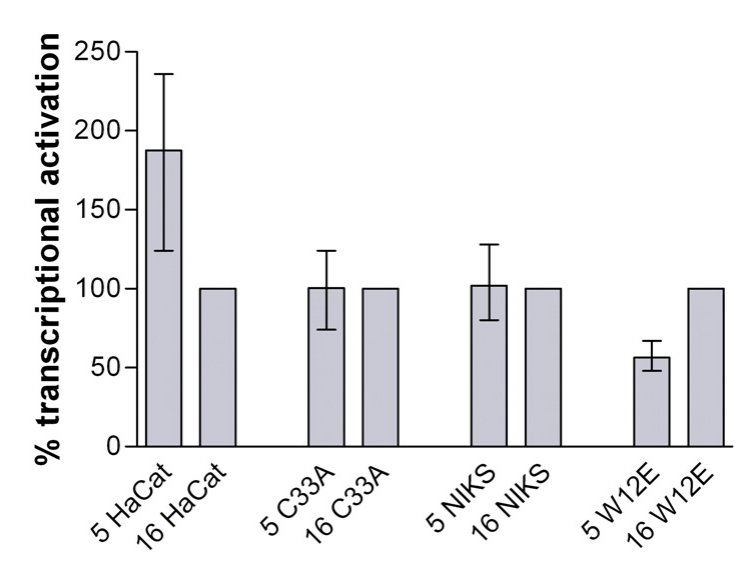
**Comparison of HPV-5 and HPV-16 LCR transcriptional activity in four different cell lines**. A pBlue-Topo vector (Invitrogen) containing the HPV-5 and HPV-16 LCR were transfected into HaCaT, C33A, NIKS and W12E cells to compare their ability to activate transcription of a beta-galactosidase reporter gene. The ability to transfect all cell lines was optimized by using a CMV promoter cloned in the same vector as the HPV LCR constructs. One day prior to transfection all cells were seeded in 24-well plates, NIKS and W12E cells were without feeder cells. All cells were transfected with plasmid DNA by FuGENE 6.0 (Roche) according to the manufacturer and protein expression was measured 3 days later. For detection the transfected cells were fixed with 1–2% formaldehyd in PBS for 5 minutes, incubated in dark with X-Gal staining buffer (2 mM MgCl_2_, 3 mM K_3_Fe(CN)_6_, 3 mM K_4_Fe(CN)_6 _and 1 mg/ml X-Gal in PBS) in room temperature up to 24 hours. Cells were washed with 5% dimethylsulfoxide in PBS and the number of blue cells were visualised and counted using a light microscope. Results were expressed as percentage of blue cells of the HPV-16 LCR transfection, which was set to 100%. Data are means +/- standard errors of the means (n = 4).

The LCR fragment was cloned from the end of the viral L1 gene to the beginning of E6. The HPV-5 LCR fragment (nt 7468-199) ended with the first ATG of the E6 open reading frame (ORF), including the suggested HPV-5 P_175 _(E6) and P_7535 _(late) promotors. The HPV-16 LCR fragment (nt 7155-103) contained the P_97 _major promoter in the beginning of the E6 gene. The correct cloning of the LCR-fragments was verified through sequence analysis. To analyse promoter activity we transfected the plasmids into HaCaT cells from adult trunk skin [21], C33A cells from the cervix [22], a spontaneously immortalized keratinocyte cell line from human foreskin NIKS (clone SG1WT) [23] and W12E (clone 20850) from a natural human cervical lesion [24]. Both the W12E and the NIKS cells contained HPV-16 episomal DNA and were cultured as described [25,26]. When we compared the efficiency of the HPV LCR constructs we found that the HPV-5 LCR activated transcription 2-fold stronger in HaCaT cells with a skin origin than either of the HPV-16 LCRs (Fig 1). The opposite was found for W12E cells from cervical mucosa. Here the HPV-16 LCR was almost 2-fold better than HPV-5 LCR in activating transcription (Fig. 1). In the other cell lines there were only small differences (Fig 1).

A factor determining HPV tropism could be viral entry into the host cell and we have found some differences in virus entry dependent on HPV type and cell origin (Mistry et al., manuscript submitted), but the anatomical tropism could not be completely explained by these results and other functional differences, like transcriptional activation, should be important for this tropism. The LCR of HPV-16 is much longer (832 bp) than HPV-5 (478 bp) and has several additional binding sites for transcription factors when compared [27]. The LCR of these two types have also been analysed *in silico *and shown to have different sets of transcription factor binding sites [4]. Common binding sites in the LCR include those for TFIID binding to canonical TATA boxes located approximately 30 bp upstream from the early start sites. Upstream of these sequences are NF-1 and AP-1 binding sites, and these are found in all HPV types studied [10]. A number of additional transcription factors shared among many HPV types have been described that bind to the LCR, e.g. Sp-1, TEF-1, Oct-1, YY1 and CDP as well as glucocorticoid responsive elements [16]. All alpha HPVs seem to have Sp-1 activated promoters, while there are no Sp-1 sites at the E6 promotors for the beta HPV-5 and HPV-8 [10]. The HPV-16 LCR also contains nuclear matrix attachment sites which could be important for controlling HPV expression [28]. HaCat cells have previously been shown to support HPV-16 LCR activation of the P_97 _promoter [10]. P_97 _seems to be the major early promoter in HPV-16 although other transcription initiator sites have been observed. Two promoters are described within the HPV-5 LCR, one just in front of the E6 gene and the other in the middle of the LCR [13]. The last is a late promoter that is up-regulated in stratum granulosum, leading to L1 and L2 expression and possibly E2 expression at low levels [13]. None of the cell lines used here was differentiated, so this region should not initiate transcription. More likely the HPV-5 LCR activation of expression was a result of an active E6 promoter. To certify this, transfections of HPV-5 LCR fragments containing either the early or the late promoter in differentiated cells could be performed. Previously, the transcriptional activity of the HPV-5 LCR fragment containing only the predicted enhancer, but lacking the 5'- and 3'-LCR, showed similar activity as the HPV-16 enhancer fragment in HaCat cells, but not in genital HeLa cells where HPV-16 was 10-fold stronger [10]. The HPV-16 LCR fragment containing the enhancer and promoter region but lacking the 5'-LCR was compared to beta HPV-8, from species 1 and both had similar transcriptional activation in HeLa cells but surprisingly HPV-16 was 2-fold stronger in HaCaT cells [10]. Our constructs contained the complete HPV-5 LCR from the end of L1 to the beginning of E6 and in HaCaT cells the transcriptional activation of HPV-5 was 2-fold stronger than HPV-16, although the levels were very low compared to the control CMV promoter we used (data not shown).

The HPV E2 protein can regulate the transcription from adjacent promoters by interacting with transcription factors like TATA-box binding protein and Sp1 [29]. E2 recognizes a palindrome sequence positioned at various sites on LCR and the alpha-papillomavirus genus has four binding sites for E2, while members of the beta-papillomavirus genus differ regarding number and positions of E2 binding sites. Two E2 binding sites are found adjacent to the E6 promoter in HPV-16, but not in HPV-5. We did not study the effect of E2, but since the NIKS and W12E cells contained episomal copies of HPV-16 we can not rule out an effect of E2 on our reporter constructs, although unlikely. To conclude, differences were found between HPV-5 and HPV-16 transcriptional activation in HaCaT and W12E cells. The LCR of HPV-5 was more effective than the LCR from HPV-16 in initiating transcription in HaCaT cells originally derived from skin and the HPV-16 LCR was more effective than the HPV-5 LCR in cervical W12E cells.

## Competing interests

The author(s) declare that they have no competing interests.

## Authors' contributions

NM participated in the design of the study, carried out molecular cloning and transfection experiments and drafted the manuscript. MS carried out molecular cloning, transfection and transcriptional activation experiments. ME conceived the study, participated in its design and coordination and helped to draft the manuscript. All authors read and approved the final manuscript.
